# The Future of Organic Insect Pest Management: Be a Better Entomologist or Pay for Someone Who Is

**DOI:** 10.3390/insects12020140

**Published:** 2021-02-07

**Authors:** David Headrick

**Affiliations:** Horticulture and Crop Science Department, California Polytechnic State University, San Luis Obispo, CA 93407, USA; dheadric@calpoly.edu

**Keywords:** National Organic Program, biology, biological control, pest management, monitoring, identification, pest control advisor, cooperative extension, education

## Abstract

**Simple Summary:**

The Federal National Organic Program (NOP) guidelines for pest management can be viewed as constraining to certified organic growers giving them a “limited toolbox” relative to non-organic crop production systems. Certifying agencies work with individual growers in setting boundaries for acceptable pest management tactics and enforce compliance for annual certification, but the knowledge required to have a successful insect pest management program can be overwhelming for growers. Traditional grower educational programs are challenged in providing the needed one-on-one training and follow up to ensure growers successfully master current and adopt newly developed pest management tactics. Gaps in the guidelines, such as monitoring, if included, could aid in grower adoption of practices that inform better decision-making and efficacy. This review promotes the idea that these issues can be overcome by utilizing experiential learning programs to educate growers and paid professionals, such as a pest control advisor. If the pest control advisor is a valued partner in the educational and extension process, they can be an effective advocate, educator, mentor, and assessor reaching more growers than education/extension programs alone, thus, achieving the NOP’s philosophical goal of a production system managed to respond to site-specific conditions.

**Abstract:**

Insect pest management in certified organic production systems presents considerable challenges for growers. The Federal National Organic Program (NOP) guidelines list acceptable tactics, but their effective use requires a considerable knowledgebase in entomology. The range of tactics allowed by the NOP are viewed as limiting by many growers and there are important elements missing from the list such as pest monitoring and identification. Educational programs must consider utilizing instructional methods and additional means of outreach that introduce new pest management tactics that are individualized, regionally appropriate and emphasize grower adoption and collaboration with local professionals. This review describes the challenges and knowledge burden associated with the listed NOP pest management guidelines, provides an educational model that includes an additional level of professional support for enhanced adoption of novel pest management tactics, or refinement of current practices, with a special emphasis on the importance of insect pest population monitoring.

## 1. Introduction

Insect pest management is one of the most challenging aspects of agricultural production that growers face. The economic success of a grower hinges on their ability to readily identify pest presence and injury levels to make informed management decisions using tactics outlined in the National Organic Program (NOP) (see Federal NOP §205.206). However, the list of acceptable practices is often described by growers as a “limited toolbox” of tactics, mainly associated with the lack of synthetic pesticides, thus making managing insect pests far more challenging than in conventional systems.

Two issues arise from the idea of a limited pest management toolbox. First, growers need to be made aware of any new advances and techniques in pest management through robust educational systems that focus on one-on-one interactions and demonstrations. Second, since the NOP guidelines serve as a point of certification and reference, they need to accurately reflect the comprehensive nature of organic pest management and specifically include pest identification and monitoring as first steps as these data inform the application and assessment of all other management tactics.

Insect pest communities in agricultural systems are dynamic and for organic growers to be successful pest managers, they must have a substantial entomological knowledgebase that is continually updated through education. Education programs need to focus on timely delivery of new pest management research and pest identification and monitoring techniques. Many organizations support the transfer of knowledge to organic growers, such as universities, extension services, non-profits, and state and federal agriculture departments. Although the goal of these agencies is to empower growers, the learning modality is typically passive listening at a workshop rather than experiential learning and assessment that ensures mastery of the information and adoption of the techniques. Further, educational programs may be infrequent or lacking for those in rural areas, making educational programming at the local level a critical priority.

These shortcomings can be overcome by having an educational model that truly trains growers and/or includes an additional level of professional support. This paper reviews the challenges and gaps related to arthropod pest management with the header section titles based on and in the order of the published pest management guidelines of the Federal NOP. This is followed by an educational model for enhanced adoption and implementation of pest management tactics, with special attention paid to the activity of monitoring for pests. This review is developed from the experience and perspectives gained by the author in ~30 years of participating in pest management practices as an educator, researcher and advisor in a variety of specialty crop production systems, and as the director of an on-campus 11-acre California Certified Organic Farmers (CCOF) farm in California.

## 2. Challenges to Organic Pest Management

### 2.1. Preventative Actions: Crop Rotation, Soil and Nutrient Management, Sanitation, and Cultural Practices to Enhance Crop Health

Preventative measures such as crop rotation, soil and nutrient management, and cultural practices are listed as first lines of defense against pests in the Federal NOP. With the exception of resistant host plants as a cultural control, these measures are indirect activities aimed at pest management. They are expected to promote the vigor of the soil and crops, resulting in less damaging impacts from pests. Crop rotation and soil and nutrient management can be listed among the few NOP elements with extensive educational guides available to growers.

Crop rotation has been shown to effectively reduce certain pest risks within specific crops [1]. Crop rotation allows growers to plan for specific management outcomes well in advance of pest outbreaks by alternating between host and non-host plants. However, not all growers have the capacity or opportunity for crop rotations due to space constraints, agronomic knowledge, their business model, the cost of leasing land to grow a no-value cover crop, or desire … they may want to grow only one type of crop. A key to the success of using crop rotation for pest management is monitoring, and in some cases, monitoring beyond the field boundaries. For example, in potato systems, crop rotations have been shown to be effective at controlling Colorado potato beetles but only when rotated fields of potatoes are at least 400 m from a field previously planted in potatoes [2]. For these growers, the need for coordinated monitoring efforts on a regional basis may achieve greater management. Although crop rotation may benefit organic growers, there are few recommendations that can be made broadly to producers, which underscores the need for farm/crop-specific monitoring data at the individual and regional level for appropriate management decisions. Regional monitoring efforts will have to be time and labor efficient and likely rely on methods other than physical collecting (nets, beat sheets) such as the use of herbivore-induced plant volatiles that attract natural enemies to a lure [3]. Coordination of regional monitoring efforts will likely be facilitated with easier monitoring tools. Depending on the situation, extension offices could serve as the principal organizing agency as pest populations will cross a variety of property boundaries.

Soil and nutrient management are long-term investments using inputs to improve certain metrics associated with a healthy soil [4]. Soil and nutrient management are the subjects of active research especially in the area of inputs that provide multiple ecological services while building soil health, such as suppressing soil pathogens. However, there are few data-based recommendations for organic growers for soil inputs that are regionally and crop appropriate that dependably aid in insect pest suppression [3,5,6,7,8,9,10,11]. Recent research activity has focused on the soil microbiome and providing inputs to enhance putative effects on above ground herbivores, but there remains very little evidence for data-based grower recommendations [10]. Soil and nutrient management require consistent monitoring and appropriate mitigations for detected deficiencies and soil monitoring/sampling activities are required by the Organic Systems Plan section G4.0 for annual certification renewal inspections [12]. Organic certifiers set the number of soil and tissue samples required from the grower before an inspection [12]. Private companies provide analytic services, that include how to take and receive the samples, results of the analyses that include deficiencies, and optional recommendations to mitigate identified deficiencies. Each sample ranges between USD 50 and USD 100 to process; however, recommendations for remediation are an additional cost per sample and can be more than twice the cost for an organic farm than a conventional farm.

Sanitation and other cultural practices have the same lack of reliable recommendation issues as with crop rotation and soil and nutrient management. For example, growers are directed by the NOP to remove disease vectors, weed seeds, and habitat for pest organisms from the growing environment. But that poses potential problems because removing habitat for pests, i.e., plant residues, also removes the habitat for natural enemies of those pests [13]. A thorough assessment of the insect community through diversity surveys could inform growers as to the impacts on insect communities before wholesale removal or tillage of crop residues is conducted, but there is a limited amount of research available for confident grower implementation and what has been published presents equivocal results [13,14,15,16].

Cultural practices that enhance crop health include the selection of plant species and varieties that are resistant to prevalent pests, weeds, and diseases. Plant resistance has been an effective insect pest management tool since its inception in the early part of the last century, but only against a limited number of pests in any given system [17]. The problem for organic growers is that the development of new insect pest resistant plant varieties will lag behind those used in non-organic systems. Organic production explicitly forbids the use of transgenic crops, which are developed more rapidly than resistant varieties from traditional breeding programs [18,19], and this is viewed as one of the constraints contributing to a limited organic grower pest management toolbox. In some cases, growers do have the opportunity to conduct their own self selection of land races for crops that show promise in resisting pests [20,21,22]. The benefit to this approach is that it meets the NOP mandate of selecting varieties with suitability to site-specific conditions. The downside is not all crops or varieties lend themselves to being propagated so easily or growers do not have the facilities to conduct such an activity. Such tactics do not take into account the diversity of cropping systems that are organically certified or the size of such operations [23]. Large-scale growers depend on nurseries or seed companies for source material and are often mono-cropped due to economy of scale [24,25]—thus, landrace selection is impractical, if not impossible. However, for some growers, educational programs can provide a better understanding of the concept of landraces and how to select and propagate resistant crop plants.

### 2.2. Mechanical and Physical Control Actions: Augmentation Biological Control, Natural Enemy Habitat Development, and Non-Synthetic Controls

#### 2.2.1. Augmentation

Augmentation biological control methods, although marginalized by Van Driesche, et al. [13] and Michaud [26], have remained popular in pest management programs and is a significant economic sector in pest management with substantial projected growth. A survey covering the years 2004–2006 determined that commercially available natural enemies comprised less than 10% of the biologically-based pest control market with an estimated gross annual value of USD 25–30 million at the wholesale level [27]. By 2018 the global biological pest control market was valued at USD 560 million and is expected to reach USD 960 million by the end of 2024 [28]. Augmentation has been a key component in arthropod pest management and insecticide use reduction in enclosed cropping systems [29,30]. However, the practice in open field settings is hampered by ecological problems [26,31] and practical problems such as the availability and quality of purchased natural enemies, access to professional consultations to ensure which species are appropriate for the problem, effective and timely deployment, and how to determine efficacy to justify costs [26,32]. The knowledge needed to implement augmentation practices that focus on a single pest/natural enemy is extensive [33,34] and only increases when considering a diversified farming system that also uses conservation biological control techniques [35].

An example of augmentation’s popularity and grower-perceived utility was observed in 2019 when California saw a surge in cannabis and hemp production. Many of these growers were new to production agriculture, and their main response to arthropod pests was a chemical application, followed by an inundative release of natural enemies [36]. However, inundation can be expensive and have inadequate results and thus growers need to have databased recommendations along with the appropriate entomological knowledge to make inundation work in an economic manner [37,38].

Augmentation by inoculation is a more effective technique than inundation but requires data on pest population field dynamics by way of monitoring to allow for timely applications. This information is often best acquired by a field representative from a biological pest management company [32]. Experience has shown that growers will apply purchased natural enemies in an inundative or calendar-scheduled manner in an attempt to reduce or overcome perceived pest problems. Growers may feel compelled to deploy natural enemies because of the human coping mechanism of “doing something is better than doing nothing” [39,40]. They may do so without economic justification, appropriate expectations, or fear of negative consequences since the use of commercial natural enemies is perceived to pose no environmental risks. Informed field representatives or other advisors can be an effective buffer to these impulses.

Growers are best served for augmentation advice by biological pest management companies that offer field representative services who participate in grower educational programs and have a strong online presence with information based on academic research and documented field experience. Application of augmentation is a multi-step process that includes monitoring pests, pest identification, knowledge of pest phenology, choosing the appropriate natural enemy species and quantity, and effective deployment. Natural enemies are also highly perishable and there needs to be considerable hands-on training to ensure the product arrives in good condition and is applied in a manner maximizing their efficacy. As such, in-person field representatives are the best means for success of augmentative biological control programs.

#### 2.2.2. Natural Enemy Habitat Development

Development of habitat on farms for the benefit of natural enemies is one of two facets of conservation biological control; the other being use of selective pesticides. In the literature, conservation biological control is known by many names: Ecological pest management (EPM) [41], Farmscaping [42], and whole-farm management [43]. The basic premise is to enhance natural enemy activity and create a crop production environment that requires far fewer inputs, as opposed to augmentation requiring consistent inputs [37,44]. There are two aspects involved in habitat development: Planting insectary plants near or within a crop to aid natural enemies, e.g., hedgerows and buffer strips [45,46,47,48], and increasing overall crop diversity within a farm, which also includes insectary plants [26,30,49]. Extensive biological and cropping system knowledge are needed for effective implementation of habitat that will lead to predictable management outcomes for both these approaches.

Insectary plantings can be with a single plant species and still provide a beneficial increase in diversity and control for a specific pest [49]. A single plant system may be more readily adopted initially by novice growers. A well-known and dependable example is the alyssum-syrphid fly-aphid system [46,47,50,51]. The use of such insectary plants may help in general to enhance the presence of certain types of natural enemies and thereby attain some overall pest management benefits [52,53,54,55], but much more research needs to be conducted to provide growers recommendations that will ensure consistent, predictable control of pests [38,44,51,56]. Research efforts also should include examination of perennial insectary plant species, as the focus of research to this point has been on annual species [47,57].

Changing the Farmscape by adding additional crop species requires new knowledge for successful growing and this may make growers hesitant in making extensive changes to plantings [45]. Seed mixes developed to support the needs of natural enemies are expensive and promoting conservation biological control by the academic community as a curative for managing chronic pest issues [26] does not take into account the willingness or ability of a grower to alter their farming system to increase plant diversity [34,35,37]. The landscape context for a farming operation can impact the efficacy of adding hedgerows or insectary plants to enhance beneficial insect diversity, including natural enemies and pollinators. A hedgerow planting in an otherwise barren surrounding landscape may have little positive effect in promoting natural enemies due to lack of a recruitment reservoir [58]. In the review by Landis et al. [59], habitat heterogeneity in and around farms had positive impacts on natural enemy activity in the studies cited, reinforcing the need to take the landscape context into account.

Conservation biological control approaches have yet to reach their full potential, but they do lend themselves to experiential educational programs conducted by the academic community that facilitate technology transfer to the grower community [23,46,47,60]. Although, for growers willing to embrace farm diversity, there must be the ability, resources, and the will to conduct in-field experimentation to achieve site-specific validation of the tactics due to differences in regional climate and crop diversity.

Other Farmscaping concepts besides conservation biological control may not be suitable or are lacking in supportive research. Ideas, such as Push-Pull technology, have been promoted for decades, but with few reasonable examples [61,62,63]. Although there are components of such systems available, the appropriate combinations and field efficacies have yet to be validated by research [62,63]. Similarly, living mulches have been shown to provide slight benefits and no detriments in organic cauliflower systems in Europe [64], but some systems show a negative effect [65]—thus, the components of different types of mulches have to be researched to show that they are a benefit to a particular cropping system [11]. The benefits and detriments of plastic mulches are still being evaluated [66,67,68] but typically have little substantial impact on managing insect pest populations [69].

Risk-averse growers may find the practices described above are difficult to enact or that they alter the way they grow to such an extent as to be impractical or undesirable [4,33,34,37]. They may also question the return on investment. If these methods were reasonable to implement and ensured a decent return on effort and expense, they would be more widely accepted and adopted. Without guidance and assurances, it is likely these avenues will remain passed over by growers opting for more reliable methods that offer broad pest management solutions with minimal farm specific pest knowledge.

#### 2.2.3. Non-Synthetic Controls

Non-synthetic controls include the use of lures, traps, and repellents. The use of lures typically involves a semiochemical or food attractant to draw in specific species or particular higher taxa (e.g., fruit flies) to entrap and kill them [70,71] but can also be used to attract natural enemies [72]. The use of lures/traps has limitations due to their target species specificity, expense, and usually requiring the cooperation of neighbors to regionally suppress pest populations to acceptable levels, unless the pest species has limited dispersal ability [73,74]. Repellents may be useful but require extensive research to make field applications reliable enough to offset the potential expense [75,76].

Lures, traps, and repellents have been developed for overall pesticide reduction and are applicable in organic cropping systems. However, as will be addressed below, growers need to be well-versed in monitoring and analyzing pest population trends, insect pest identification, and degree day estimations to make these approaches work effectively and economically.

Pheromone mating disruption has been used successfully for lepidopteran pests in agriculture for decades and the examples are legion [77,78,79,80]. However, there are far fewer pheromone mating disruption applications for other taxa, often due to biological incompatibility such as asexually reproducing species or asexual generations within a species [81]. Investigations of using sex pheromones in other taxa should be encouraged for future organic pest management as this tactic is a powerful population regulation tool that can significantly reduce pesticide use [23], but monitoring needs to be conducted to ensure proper timing, trap placement, and follow-up for efficacy.

### 2.3. Chemical Controls: Biological, Botanical, or Approved Synthetic Substances

The challenges with pesticide use are well-known: Worker and environmental safety, insecticide resistance, safe storage and handling, adherence to label requirements, use reporting, accurate applications, residues, pollinator poisoning, and marketing issues [82,83]. Some of these same issues apply to organic pesticides as well, but less so due to lower toxicities [84] and fewer residue issues [85].

The following highlights three inter-related issues to pesticide use in organic cropping systems that will impact future chemical pest management practices: The type of products available and ultimately adopted, economics, and when to use them.

Despite the best efforts at prevention and mechanical/physical control, pest problems will occur and growers are told to use chemicals only as a last resort. The Federal NOP does not explicitly state, but implies, that chemical pesticides be chosen to prevent environmental harm, which is another facet of conservation biological control—the use of selective chemicals. These chemicals are physically or physiologically selective to specifically preserve currently active natural enemies from harm. Ironically, in spite of the NOP’s emphasis on prevention, Goldberger and Lehrer [33] validated the work of earlier studies [34,35] that showed growers more readily adopted the tactics of reducing harm to natural enemies by using selective pesticides rather than adopting the practices of beneficial habitat development or even augmentation due to their familiarity with the pesticide application process and rapid tangible results.

Once the need to apply a chemical has been determined, a decision must be made for the most effective, economical and least disruptive chemical that can be integrated into the system. This is a crucial decision. Naranjo et al., [86] showed that growers from all over the world were willing to pay much higher prices for pesticide products that were safer for beneficial insects. However, growers need to know that the extra expense results in economic and environmental benefits and will effectively manage pests. They also need to know that the application will not disrupt other pest management practices, especially protecting natural enemies. There are few readily available guides for growers attempting to simultaneously integrate chemical and biological tactics. Koppert, Inc. [87] and Biobest [88] have developed open access natural enemy/pesticide compatibility guides available online and as mobile apps, however, these guides provide information that is often limited to the company’s respective product lines.

The current products of choice for organic growers are typically botanical or microbial pesticides, a growing and diversifying market [38,89,90,91,92]. But the adoption of microbial pesticides will be biased towards those that are applied and act like traditional pesticides, such as microbial Bt [90]. There are few studies that show what types of insecticides organic growers currently use in terms of frequency and amount. The cannabis survey discussed above, which was not based solely on organic production but did include reduced risk chemical approaches, showed that “microbials” were the chemical category of choice for growers with “unknown organic products,” azadirachtin, and oils rounding out the top four insecticidal products [36]. Entomopathogenic nematodes (EPN) have also become popular as an equivalent to a chemical control attack on pests because of their unique biology and ease of application. In some cases, EPN can be applied through traditional pesticide application or irrigation equipment, potentially facilitating their adoption by growers. However, organic growers face technical challenges because many commercial formulations of EPN are stabilized with fungicides that are not approved by the NOP or Organic Materials Review Institute [93].

Growers want to use chemical pesticides when they feel the need arises [94]. They may work at preventative measures, but growers will use chemicals in response to any perceived economic pest threat and not as a last resort. As much as academics want growers to adopt various ecologically based pest management approaches, growers need to know that their investment and income can be protected from pests at a moment’s notice. Others argue that the suite of chemical options should be broadened for organic growers with mounting evidence that organic pesticides are ineffective, resulting in more applications, are harmful in other ways to the environment, have non-target issues, and are not imparting the perceived health benefits when compared to conventionally grown crops [95,96,97]. McGuire [97] specifically argues that banned synthetic pesticides reduces avenues for enhancing organic pest management. This again points to the constraints on the grower toolbox.

### 2.4. What Is Missing from the Federal NOP? Monitoring and Identification

The NOP does not mention monitoring, and concomitant pest identification, as part of pest management but these activities should be explicitly listed among the first preventative tactics so that they filter through the entire certifying/educational infrastructure to the grower. For many pest management control tactics, a robust monitoring system is required for a grower to determine if an application is warranted and to time it with confidence. Insect pest population assessment, specifically determining the presence, density and dispersal of a variety of insect pest species and their natural enemies is a daunting aspect to pest management for growers and advisors alike [98], but with robust benefits (Table 1).

#### 2.4.1. Monitoring

Monitoring or scouting is a time-consuming constraint on growers due to the need for consistency and follow up to determine treatment efficacy [98]. Monitoring a cropping system requires extensive knowledge of weeds, pathogens, arthropods and vertebrates. For diverse cropping systems the knowledge burden is compounded by the number of different crops. The benefits of consistent monitoring allow for early detection that is necessary to time and select pest management actions, especially for tactics slow to develop, such as inoculation augmentation biological control, or conservation biological control tactics, such as insectary plantings [59]. Determining if an insect pest population represents an economic concern is an additional layer of complexity that requires current and historical knowledge [99]. Assessing economic injury levels and action thresholds require accurate, consistent field information [100] and most growers rely on “nominal thresholds” [101] that are based on a grower’s experience rather than a formal economic injury level calculation [102]. The lack of meaningful thresholds for most pests and cropping systems is a well-known gap that can be bridged by consistent monitoring, but the results of such monitoring need to be a part of the annual certification process to ultimately develop appropriate thresholds for individual farmers.

#### 2.4.2. Identification

Identification is the key first step in the development of a pest management plan for any type of growing situation [99]. There are very few publications indicating the extent to which growers struggle with identification of plant pests. Piñero and Keay [103] conducted a grower survey in the state of Missouri where 84 conventional and organic growers self-rated their ability to identify pests giving themselves an average of 3.14 out of 5-point rating system; there was no quantified assessment regarding their actual accuracy.

Pest management products and methods that are taxa-specific and stage-specific require accurate identification for effective use, requiring growers identify pests to at least the taxonomic level of order. For example, certain Bt products target Lepidoptera, additionally, these products are typically more effective on early instars [104,105,106] and so also require knowledge of stage class distributions of in-field pest populations. Species-level identification is necessary for literature searches, applying data-based recommendations, and especially for selecting appropriate natural enemy species for biological control programs [107].

Much like monitoring, identification requires extensive knowledge for a daunting number of taxa. The process to achieving a taxonomic identification involves a hierarchy of expertise. If the grower or advisor/field representative is familiar enough with the pest, they can identify it on their own. If not, they may confer with peers to settle on an identification determination. Through experience, growers and advisors learn the common, predictable pests and anticipate them accordingly. But infrequent or unusual pests do show up in fields regularly, in which case, identification efforts move up to county departments of agriculture or extension office personnel. If the county personnel are not trained or certified to make an identification on a particular taxon, then those specimens are sent to the state agriculture department for identification. In some cases, federal USDA ARS or university expert identifications are sought, but can take considerable time and so hamper the immediacy of in-field problem solving.

There is little published literature concerning increasing the capacity of growers and advisors to identify plant pests and diseases, especially new arrivals. Levy [108], Bagamba et al. [109], and Yang et al. [110] indicated that the ability of growers and industry personnel to identify insects was increased when training was provided during intensive educational outreach programs [111]. However, such training efforts are largely limited in scope to a specific crop, or specific suite of crop pests, and many organic growers incorporating other NOP standards that encourage crop diversification are at a distinct disadvantage in these educational settings because of the diversity of insects they may encounter in their own operations.

The advancements in cellphone cameras have aided grower-level identification by making it easier to send photos to peers, advisors, or other professionals for an ID. Use of online resources are also available, e.g., bugguide.net, inaturalist.org. Identifications from online resources are from both professionals and amateurs alike but accuracy of an identification can be unreliable from some online sources and/or may be misinterpreted by the lay public.

App-based monitoring and identification tools will continue to improve, but academic oversight should be a priority for their development and subsequent training in educational pest management programs. Some institutions have compiled lists of apps with basic descriptions [112] but no serious vetting. The limitations of these apps are that they cost or require an account, they tend to focus on one crop such as corn or strawberries, and they do not provide tools for population analyses to aid in pest management decisions. If recommendations are provided for crop-specific pest problems, the resulting information is either from an already established institutional website or has no affiliation and may be suspect. In the long-term, developers of these apps may not remain in business, provide sufficient updates or adequate customer service. Thus, it is reasonable to look to land grant colleges and universities to develop apps to provide growers with trustworthy, no-cost, ad-free, data-driven, and tested tools suitable for their crops/region to aid in individual management decisions.

### 2.5. Other Factors

#### 2.5.1. Biological Knowledge

All growers face the challenge of having enough biological knowledge to properly use certain pest management methods. Biological control is a knowledge-intensive management approach whose users benefit from having a strong entomological background to ensure successful implementation [31,33]. Predator/prey population regulation dynamics, with their inherent lags, is a fundamental concept for successful manipulation of biological control agents in augmentation programs [13,44,113]. Understanding the basics of insect pheromone biology and degree days is required for effective timing of mating disruption programs or applications of natural enemies, pathogens, microbials, or other approved insecticides against a particular life stage, such as codling moth [114,115].

#### 2.5.2. External Factors

Organic growers, as do any growers, face significant and potentially devastating economic challenges due to invasive species and subsequent government quarantine mandates [116]. It is not clear how well informed and up to date certifying agencies are regarding invasive species. Impacts from a pest invasion can be abrupt and disruptive to the marketing of the current crop, or worse, a call for crop destruction. If non-approved pesticides or other non-certified eradication tools are required for invasive species eradication efforts on or near the crop, the organic grower may lose certification for all or part of a harvest, such was the case with light brown apple moth [117] and Asian citrus psyllid [118]; but rarely does the farm lose its certification and have to restart the three-year recertification process [119]. Timely information and strong public relations campaigns are crucial for the academic community to engage the public in invasive species programs. Growers, and the public, have a heightened sense of alarm and anxiety associated with the impacts of invasive species and programs such as the California Citrus Threat [120] serves as a model system for this particular challenge.

Organic growers also face issues due to migratory insect pests or intrusions of off-site GMO crop elements such as pollen. Migratory species like whiteflies, thrips, and diamondback moth, can move suddenly and unexpectedly into an area changing the pest dynamics for a crop literally overnight. Other consequences resulting from an invasion of a migratory species is that growers inherit populations that may have developed pesticide resistance, thus rendering weaker organic insecticides with the same mode of action useless. Understanding resistance management and paying attention to the Insecticide Resistance Action Committee (IRAC) [121] mode of action numbering system is one that all growers should be made aware of for a comprehensive pest management plan [122].

Finally, the impacts of climate change on pest populations are current and dynamic. Pest populations are starting earlier, staying longer, and developing through more generations [123,124], thus impacting monitoring practices and timing of cultural and other pest management tactics.

## 3. Overcoming Challenges and Looking to the Future

All told, the knowledge burden is increasing, and the grower’s need for information and validation for taking a particular pest management action are getting more intense. After reviewing these challenges and contemplating how to move forward, a quote from Dr. Joseph Morse, Emeritus Entomologist, UC Riverside, serves as a guiding principle. He stated to the growers and academics at a California Citrus Research Board grower education meeting in 2003 that, “Either growers will become better biologists, or they’ll need to pay someone who is.”

The focus for the next section will attempt to lay the framework for an education-based system for empowering growers and their advisors to be better biologists, or specifically for this review, better entomologists, in their pest management efforts. Achieving this goal will require creating free or affordable access to meaningful, regionally appropriate educational programs from academics and individualized on-farm advising through the use of a model similar to the California Pest Control Advisor’s (PCA) program [125].

**Figure 1:**
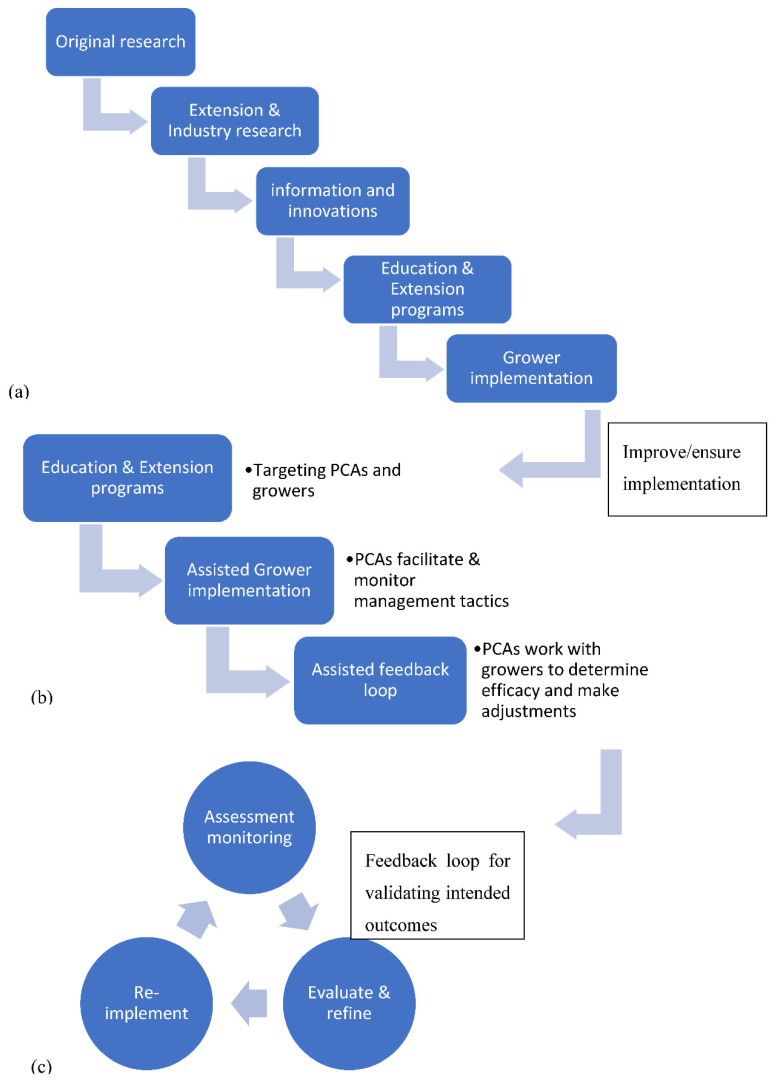
An illustration of pest management information flow. (**a**) Information typically flows from original research to extension and private industry research to innovations/modifications in pest management tactics to the end user by way of educational programs conducted by academics, extension agents, state and local agriculture departments, non-profits, private industry, professional societies. (**b**) Effective grower implementation is uncertain and can be improved/aided with additional professional guidance from a Pest Control Advisor (PCA). Ensuring the PCA is included in the educational process enhances their knowledgebase and consistent messaging to growers. (**c**) Finally, the PCA provides the necessary skillset of monitoring for effective outcomes and advice on improvements that feeds back into future implementation.

The typical transfer of information to growers begins with academic experts conducting research through a series of five steps referred to as the Atwater Directives [126]. The final step is where the Cooperative Extension Service steps in to enhance and impart research findings to the growers; a model with a long history of success Figure 1 [127]. These services are state-funded and provide information free of charge. However, cooperative extension budget reductions have made it nearly impossible for the extension service to provide the full number of individualized, hands-on advising and follow-up visits that are needed for today’s grower community [128].

In California, the reduction of the cooperative extension program has been buffered by the Department of Pesticide Regulation’s PCA licensing program and grower reliance on PCAs for pest management information and advice has continued to grow since its inception [129]. In a recent law review, Vanzant [130] listed the following statistics for PCA reliance. In 1983, 75% of a large survey of tomato growers ranked PCAs as their “most important source of pest control information”; in 2000, a survey of 453 almond growers in the San Joaquin and Sacramento Valleys revealed that 97% of those growers relied on PCAs for advice regarding pest management; and in 2007, a survey of 266 California cotton growers showed that 99% of those growers relied on PCAs for their pest management needs. In a more recent study, Goldberger and Lehrer [33] showed PCAs and chemical company field representatives were the primary source of pest management information for walnut and pear growers in the Pacific northwest.

These reports indicate a line of information transfer from the researcher to the grower that includes the PCA as a key information and recommendation resource for growers. The role of the PCA as a source of information has been acknowledged by the Department of Pesticide Regulation as they require 40 h of continuing education every two years to maintain the PCA license. The educational programs that are designed and delivered by the academic community for CEUs in California focus on having the PCA as part of the audience and in some cases as presenters. In other states, Certified Crop Advisors (CCA) or graduates of Plant Doctor programs (University of Nebraska-Lincoln and University of Florida) play a similar role to the PCA. Thus, licensed professionals such as PCAs do the leg work and develop management plans that are then discussed with individual growers.

Organic growers are able to utilize the services of PCAs, and California and Arizona require a PCA recommendation for certain microbial pesticides. They also offer advice regarding the use of natural enemies and cultural controls to tailor university-derived research to the specific crop/region and available resources to best serve the grower [131,132]. Ehler and Botrell [133] termed it “supervised” help for pest management.

If pest management information transfer were to include a licensed professional such as a PCA whom the grower will pay to be the better entomologist, then that idea needs to be tempered with the fact that there will be a conflict of interest for PCAs that are employed by pesticide distribution companies [130,133]. It will be critical for growers to ensure that they hire independent PCAs for objective information but also have the choice to use company representatives for specific product recommendations.

The use of social media is having an impactful role in the transfer of information to and among growers. Social media is a tool that provides valuable opportunities, but also spreads misinformation quickly and broadly. There are no studies that currently quantify the reliance that growers have on social media platforms for pest management information, but it’s importance and ubiquity in pest management information exchange has been discussed in detail by Holt et al. [134]. Social media can be effective in information transfer [135,136] but possibilities of abuse and misinformation make it a challenge to ensure growers can distinguish between evidence-based reliable information and inaccurate or misleading information. Solis-Toapanta et al. [137] recently conducted a fascinating study of Reddit threads involving pest management information exchange; the amount of misinformation is not inconsequential. This should be carefully considered because growers have stated that the importance of information obtained from other growers was at times on par with, or in some instances more important than, university scientists and extension personnel [33].

Ultimately, with a PCA-included educational model, university-derived education programs become “train the trainer” programs with significant benefits [128]. The benefit to the grower is having both extension personnel and paid professionals with a consistent message. As stated by Baker et al., [38] education that follows appropriate research is a key to successful technology transfer [122]. The benefit for the organic grower will be professional advisors with an enhanced knowledgebase related to organic pest management tactics, such as Farmscaping, augmentation biological control, and microbial pesticides, and will likely amplify their adoption by growers due to the well-established relationship of most growers having PCAs as their preferred/primary source of such information [33,130].

The mechanisms of information transfer to the grower envisioned here would be through site visits with individualized explanations of concepts using visual graphics, then revisiting to determine the efficacy of tactics and finally a feedback loop for assessment of the education process itself. If the process works, the grower will see the results; in other words, their assessment will be measured as the net return after harvest. For the PCA, the assessment will be based on the success of the grower relationship and its continuation. For the academics, especially the extension agents, there must be an accounting to close the loop—using metrics and assessment of impacts that are not just “number of attendees” but actual documented in-field successes such as yield increases, reduced pesticide applications, areawide adoption of reduced-risk management tactics, etc.

### An Example of Educational Programming for Improved Pest Management for Organic Growers

As mentioned throughout, monitoring is an area in need of strong emphasis in educational programs for professional advisors and growers. Monitoring also serves as an example of where the PCA role can be crucial in empowering growers and working collaboratively with them on decision-making. Development of monitoring programs that are adaptable to specific growing situations is key. Advisors need to have tactics that are easily implemented for assessing the density and distribution of pest populations and that allow for grower participation. Having the results of a pest monitoring program be a required part of the annual certification process could establish and normalize the activity, as is the case for soil sampling.

Key elements for teaching monitoring include but are not limited to the following:

1. Identification of pest species, appropriate field sampling patterns, determination of the number of samples needed, recognition of life stages, and what to count [138].
2. Record keeping of scouting data includes developing a database and analysis tools that helps growers visually interpret their findings and provide easily retrievable information of the current season and past years.
3. The use of electronic capture of real time field scouting data via apps on tablets and phones must be included as well. Such technology has already been instituted by many professional companies, but these are proprietary. Although there is a proliferation of farm aiding apps available for android and iPhones, there is little vetting for growers to ensure they are getting a quality product, maintaining privacy, or filtering unwanted influences on decision making as mentioned above. Most importantly such apps need to have a graphing tool to chart, in real time, population trends including natural enemy population dynamics such as seen with the iPM app [139].
4. Information on multi-trophic interactions in diverse systems that includes host plant ranges of key pests and their associated natural enemies, designating those that are generalists and specialists and possible crossovers among crop plant species.
5. The use of qualitative rating systems is an excellent approach to capturing a variety of pest impacts that is easily taught and widely applicable across crops, pest species and not limited by the size of the operation. Some examples of its use include Capinera et al. [140], Bellows et al. [141], Schroeder et al. [142], Ward [143], and Brainard et al. [144].
6. Predicting pest occurrences and subsequent in-field population dynamics is crucial to cost-effective pest monitoring. As an example, Kogan et al. [145] outlined the successional colonization of annual crops by herbivores and natural enemies as a means to help predict certain pest groups in crops that have their succession clock restarted with every new planting. This concept may serve as a general guide to help growers better predict, time and prevent certain types of pest population issues such as seen with pests in nut crops [146].
7. Understanding degree days also is crucial in preventative/predictive pest management planning. The University of California’s Integrated Pest Management website has a degree day calculator that is region, crop and pest specific [147]. This interface helps growers determine heat unit accumulation for crop phenology and prediction of pest growth and development. Additionally, degree day accumulation prompts certain management actions, growers benefit from programs that include timely reminders such as seen with Washington State University’s Decision Aid System [148]. However, education programs must teach advisors and growers how to validate these models in their area to fine tune the predictive ability, thus, making the tool more useful for an individualized regional approach if a Decision Aid System is lacking for their locality.

Pursuing the educational programs described here makes the entire system work better with growers taking an active and informed role in the discussions with their advisors and extension agents about their crop and how best to protect it. It allows for ownership and personal responsibility in the outcomes. Reinforcing the benefits of monitoring, or any subject, through educational channels is key to adoption as growers consistently weigh cost/benefit outcomes in their daily activities and business models [38,122].

## 4. Conclusions

This review is based on the issues that the Federal NOP guidelines for pest management can be viewed as constraining to certified organic growers in their attempts at economically successful management of a variety of pest situations; that the knowledge required, especially entomology, to successfully implement current management tactics is overwhelming; and that there are significant gaps in the guidelines that, if resolved, could aid in grower adoption of practices that inform better decision making and efficacy.

The guidelines were never intended to be comprehensive and thus their generality leaves room for interpretation. Certifying agencies work with individual growers in setting boundaries for what are acceptable tactics for pest management and then enforce compliance for annual certification. However, this does not alleviate what is considered a limited toolbox relative to non-organic crop production systems, nor does it provide the necessary training to effectively implement currently established or new tactics.

The pest/cropping system knowledgebase needed to have an economically successful and sustainable pest management program is daunting. Traditional grower educational programs through agencies such as universities, extension services, non-profits, and state and federal agriculture departments are challenged due to budget and personnel reductions, to provide the needed one-on-one training and follow up to ensure growers successfully master current and adopt newly developed pest management tactics.

One of the most significant gaps in the NOP guidelines is the lack of pest monitoring and identification. The benefits of monitoring are well established—early detection of pest populations, determination of location and density, establishing growth trends, having a retrievable historical record, proper timing of cultural, biological and chemical management tactics, and follow-up assessment of efficacy. Including monitoring and identification in the crop pest, weed, and disease management practice standard will serve to elevate the importance of this activity, making it enforceable for certification and eventually normalizing its use.

This review promotes the idea that these issues can be overcome by utilizing experiential learning programs to educate and empower growers and paid professionals, such as a PCA and further having PCAs provide hands on grower guidance. Funding for these programs could be based on two models familiar to citrus growers: the California Citrus Research Board’s grower education program [149] and the Fillmore Protective District [150,151]. Both models are funded through bin taxes on harvested fruit, thus oversight and buy-in to programs are prioritized within the grower community. If the PCA is regarded as a valued partner in the educational and extension process, they can be an effective advocate, educator, mentor, and assessor for growers and ultimately reach more growers and ensure effective adoption and use of a variety of management tactics. Thus, achieving the NOP’s philosophical goal of a production system managed to respond to site-specific conditions by integrating cultural, biological and mechanical practices.

## Figures and Tables

**Table 1:** Consistent monitoring is crucial to all pest management decisions and to inform future adjustments to ensure effective and economically viable outcomes.

Benefits of Monitoring:
1. Early detection of pest populations
2. Determination of location and density
3. Establishing growth trends
4. Having a retrievable historical record
5. Proper timing of cultural, biological and chemical management tactics
6. Follow-up assessment of efficacy

## Data Availability

Not applicable.
